# Naringenin inhibited vascular calcification and attenuated senescence-associated changes through the p53/TOP2Aaxis

**DOI:** 10.3389/fphar.2026.1785797

**Published:** 2026-05-08

**Authors:** Xiaoya Tong, Meixian Zhao, Ying Hu, Qian Zhang, Rong He, Rui Yan

**Affiliations:** 1 Department of Nephrology, The Affiliated Hospital of Guizhou Medical University, Guizhou Medical University, Guiyang, China; 2 Department of Nephrology, Guizhou Provincial People’s Hospital, Guiyang, China; 3 NHC Key Laboratory of Pulmonary Immunological Disease, Guizhou Provincial People’s Hospital, Guiyang, China; 4 Guizhou Provincial Key Laboratory of Pathogenesis and Prevention of Common Chronic Diseases Research, Guizhou Provincial People’s Hospital, Guiyang, China; 5 Guizhou University of Traditional Chinese Medicine, Guiyang, Guizhou, China

**Keywords:** chronic kidney disease, naringenin, p53/TOP2A signaling axis, senescence-associated changes, vascular calcification

## Abstract

**Background:**

Vascular calcification (VC) is a major cardiovascular complication of chronic kidney disease (CKD). Senescence-associated vascular changes have been increasingly implicated in CKD-related VC. Naringenin, a natural flavonoid with anti-oxidative and anti-inflammatory properties, has shown protective effects in several age-related disorders, but its role and mechanism in CKD-associated VC remain unclear.

**Methods:**

Human vascular specimens, high-Pi-induced vascular smooth muscle cells (VSMCs), and an adenine/high-phosphate-induced rat model of CKD-associated VC were used to evaluate the relationship between calcification and senescence-associated changes and to assess the effects of naringenin. Histological staining, SA-β-Gal staining, Western blotting, immunofluorescence, immunohistochemistry, RNA sequencing, molecular docking, chromatin immunoprecipitation, and dual-luciferase assays were performed to explore the possible mechanism.

**Results:**

VC was positively associated with senescence-associated changes in patient tissues, cultured VSMCs, and rat aortas. Naringenin significantly reduced calcium deposition and attenuated senescence-associated marker changes both *in vitro* and *in vivo*. Transcriptomic analysis implicated the p53 pathway and identified TOP2A as a downstream effector associated with naringenin treatment. Mechanistically, naringenin was associated with suppression of p53 signaling, relief of p53-mediated transcriptional repression of TOP2A, and restoration of TOP2A expression. Functional assays further suggested that p53 activation or TOP2A knockdown attenuated the protective effects of naringenin.

**Conclusion:**

Naringenin inhibits vascular calcification by modulating the p53/TOP2A axis, and this protective effect is accompanied by attenuation of senescence-associated changes. These findings support naringenin as a mechanistically relevant candidate for CKD-associated vascular calcification, although the upstream mechanism underlying p53 suppression remains to be clarified.

## 1 Introduction

Worldwide, kidney disease affects roughly one billion people, with many cases progressing to chronic kidney disease (CKD) (KDIGO, 2024). CKD represents a significant global health burden (Jadoul et al., 2024; Bello et al., 2024). It is strongly linked to cardiovascular disease (CVD), and vascular calcification (VC) in CKD patients is recognized as a powerful predictor of CVD risk (Zoccali et al., 2023). The diagnosis of VC in patients with CKD remains a considerable challenge, largely due to its complexity, which involves multiple pathological mechanisms in the early stages (Hutcheson and Goettsch, 2023). Promoting factors of VC include mineral imbalance (Mathew et al., 2017), in which elevated phosphate and calcium concentrations accelerate VC by inducing osteogenic or chondrogenic differentiation, extracellular vesicle release, apoptosis, and extracellular matrix (ECM) degradation (Bäck and Michel, 2021).

Meanwhile, the intricate interplay between CKD and VC is increasingly linked to senescence-associated changes (Sutton et al., 2023). In CKD, factors such as uremic toxins, persistent inflammation, and oxidative stress synergistically speed up vascular aging (Speer et al., 2022; Levstek and Trebušak Podkrajšek, 2023). Beyond VSMC osteogenic transdifferentiation, CKD-related VC also involves cell-cycle arrest pathways mediated by p53/p21 and p16/RB signaling (Fang et al., 2024). This premature vascular aging is characterized by heightened expression of senescence-associated markers (Tölle et al., 2022). Notably, therapeutic strategies targeting senescence-associated pathways have shown promise: for example, the combination of dasatinib and quercetin significantly reduced aortic senescence markers and calcification in animal models of aging and atherosclerosis, suggesting that targeting senescence-associated pathways could be a novel approach to treat CKD-related VC (Roos et al., 2016).

Naringenin is a naturally occurring citrus flavonoid abundantly found in grapefruits and other citrus fruits (Ribeiro, 2011). It exhibits multiple biological activities including anti-inflammatory, antioxidant, and lipid-lowering effects making it a compelling candidate for combating age-related vascular disorders such as VC (Rivoira et al., 2021). Previous studies demonstrated that naringenin inhibits VSMC calcification and attenuates vascular stiffness by reducing inflammatory responses and oxidative stress (Li et al., 2023). Furthermore, naringenin has been reported to modulate senescence-associated pathways through activation of the SIRT1 signaling pathway (Du et al., 2009).

Another critical regulator of vascular stress responses is the tumor suppressor protein p53. In response to DNA damage or oxidative stress. p53 is activated and promotes cell-cycle arrest and stress-associated phenotypic changes (Fang et al., 2024; Li et al., 2024). Importantly, DNA topoisomerase IIα (TOP2A) – a downstream target of p53 and a key enzyme controlling DNA topology – plays a pivotal role in cell proliferation and vascular aging. TOP2A is normally highly expressed in proliferating cells, where it facilitates DNA replication and proper chromosome segregation (Zhang et al., 2025). In stressed vascular cells, however, the p53/p21 and p16/RB pathways converge via the DREAM complex to repress numerous cell-cycle and DNA repair genes, including TOP2A (Kandhaya-Pillai et al., 2023).

In the present study, we investigated whether naringenin could attenuate vascular calcification and accompanying senescence-associated changes in CKD-related VC and explored the possible involvement of the p53/TOP2A axis. Our findings suggest that naringenin exerts anti-calcific effects in this context and support the p53/TOP2A pathway as a mechanistically relevant signaling axis.

## 2 Materials and methods

### 2.1 Chemicals

Naringenin (N5893) and Alizarin Red S (TMS-008) were purchased from Sigma-Aldrich (St. Louis, MO, United States). β-Glycerophosphate (IG1340) and L-ascorbic acid (SV8180) were from Solarbio (Beijing, China), while dexamethasone (D917754) was obtained from Macklin (Shanghai, China). All reagents were of analytical grade; DMSO was used as solvent when required, and its final concentration did not exceed 0.1%.

### 2.2 Human tissue specimens

Vascular specimens were obtained from CKD5 patients receiving maintenance blood purification therapy at Guizhou Provincial People’s Hospital. Radial artery samples were collected during forearm autologous AVF creation. Written informed consent was obtained from all participants. The study was approved by the Ethics Committee of Guizhou Provincial People’s Hospital (Approval No. [2023]096) and conducted in accordance with the Declaration of Helsinki. Specimens were fixed, paraffin-embedded, and sectioned at 3 μm for subsequent analyses.

### 2.3 Histological staining and calcification scoring of human vascular specimens

Calcium deposition in human vascular specimens was assessed by Von Kossa and Alizarin Red S (ARS) staining. Sections were independently scored by two blinded investigators using a 0–3 semiquantitative system, and discrepant cases were resolved by joint review. Specimens were classified as non-calcified (score 0), lightly calcified (scores 1–2), or heavily calcified (score 3).

### 2.4 Bioinformatics analysis

To explore molecular signatures associated with calcification, the GEO dataset GSE104140 was analyzed (https://www.ncbi.nlm.nih.gov/geo/). Differentially expressed genes (DEGs) were identified using thresholds of p ≤ 0.05 and |log_2_FC| ≥ 1. Gene signatures were visualized by volcano plots and heatmaps. Candidate small-molecule modulators were identified through the Connectivity Map (CMap, https://clue.io/). Binding motifs of transcription factors were further predicted using JASPAR (https://jaspar.genereg.net/analysis).

### 2.5 Cell culture and calcification model

Human vascular smooth muscle cells (HVSMCs) were purchased from Fenghui Biotechnology (Catalog No. FHHUM151). Cells were revived and cultured according to the supplier’s instructions and maintained in DMEM supplemented with 10% fetal bovine serum and 1% penicillin–streptomycin at 37 °C in a humidified incubator with 5% CO_2_.

### 2.6 Calcification induction and naringenin treatment *in vitro*

At 70%–80% confluence, HVSMCs were cultured in calcification-inducing medium containing inorganic phosphate (Pi; 2.6–3.0 mM), ascorbic acid, and dexamethasone, while control cells were maintained in regular medium. The medium was changed every 48 h, and cells were collected at the indicated time points according to the downstream assay in parallel time-course experiments. For naringenin treatment, cells were exposed to 25 μM naringenin or an equal volume of DMSO simultaneously with calcification induction, with DMSO kept ≤0.1% and identical among groups. Naringenin or vehicle was replenished at each medium change. DMSO was used for *in vitro* stock preparation, whereas 0.5% CMC-Na was used for *in vivo* gavage.

### 2.7 RNA extraction and qPCR

Total RNA was isolated using TRIzol™ and quantified by NanoDrop. Reverse transcription was performed using the TransScript® Two-Step RT-PCR kit, and qPCR was conducted with SYBR Green chemistry. Relative expression was calculated by the 2^-ΔΔCt method. Primer sequences are provided in Supplementary Table S1.

### 2.8 Gene perturbation

Transient transfection was carried out using Lipofectamine 3,000. Gene expression was silenced with siRNA/shRNA, or enhanced via overexpression plasmids. Details are summarized in Supplementary Table S2. Efficiency was validated before subsequent analyses.

### 2.9 Western blotting

Proteins were extracted with RIPA buffer containing inhibitors, quantified by BCA assay, separated by SDS–PAGE, and transferred to PVDF membranes. Membranes were blocked and incubated with primary antibodies (e.g., p53, p21, γ-H2AX, TOP2A, Runx2, BMP2, Lamin B1), followed by HRP-conjugated secondaries. Signals were visualized by chemiluminescence and quantified densitometrically (antibody details in Supplementary Table S3).

### 2.10 Immunohistochemistry

Paraffin-embedded aortic sections were deparaffinized, rehydrated, and subjected to antigen retrieval. Endogenous peroxidase activity and nonspecific binding were blocked before overnight incubation at 4 °C with primary antibodies against p53, TOP2A, p21, and Lamin B1, followed by HRP-conjugated secondary antibodies. Signals were visualized with DAB, counterstained with hematoxylin, and imaged under identical microscope settings.

### 2.11 Alizarin Red S staining

Cells were fixed with 4% paraformaldehyde for 20 min and stained with 1% Alizarin Red S (pH 4.2) for 30 min at room temperature. Mineralized nodules were imaged microscopically and quantified as calcified area relative to total area.

### 2.12 Von Kossa staining

Aortic sections were treated with 5% silver nitrate and exposed to UV until calcium deposits appeared dark brown/black. Unreacted silver was removed with 5% sodium thiosulfate. Sections were counterstained, and calcified lesion area was quantified as a percentage of medial area.

### 2.13 SA-β-galactosidase assay

Cellular senescence was assessed using a Senescence β-Galactosidase Staining Kit according to the manufacturer’s instructions. At the indicated time points, cells were fixed, incubated with freshly prepared SA-β-Gal staining solution at 37 °C overnight in a CO_2_-free incubator, and examined under a light microscope. Cells showing obvious blue cytoplasmic staining were considered SA-β-Gal-positive senescent cells. For quantification, at least 5 randomly selected non-overlapping fields were analyzed per sample at the same magnification, and the percentage of positive cells was calculated.

### 2.14 Immunofluorescence

Cells on coverslips were fixed, permeabilized, blocked, and incubated with primary antibodies, followed by fluorophore-conjugated secondaries. Nuclei were counterstained with DAPI. Images were acquired with a Zeiss confocal microscope; fluorescence intensity and localization were quantified under consistent acquisition parameters.

### 2.15 Animal experiments

Male Sprague–Dawley rats (8 weeks old, 180–220 g) were housed under SPF conditions and randomly assigned to control, CKD-VC, and CKD-VC + naringenin groups after 1 week of acclimatization. All procedures were approved by the institutional animal ethics committee (Approval No. 1240221). CKD-associated vascular calcification was induced by feeding rats 0.75% adenine plus 1.5% high-phosphate diet from week 0 to week 4, followed by 0.25% adenine plus 1.5% high-phosphate diet from week 5 to week 8. Naringenin was administered by oral gavage at 100 mg/kg/day (10 mL/kg in 0.5% CMC-Na) from week 5 to week 8, while control and CKD-VC rats received vehicle. At the end of week 8, thoracic and abdominal aortas were collected for histological and molecular analyses.

### 2.16 RNA sequencing

Total RNA from aortic tissues of the CKD-VC and CKD-VC + naringenin groups (n = 3 per group) was subjected to library construction and sequencing on the Illumina NovaSeq 6,000 platform. Differentially expressed genes were identified after quality control and normalization using thresholds of |log_2_ fold change| > 1 and p < 0.05. KEGG and GO analyses were performed for functional enrichment. Given the limited sample size, RNA-seq was used as an exploratory transcriptomic analysis.

### 2.17 Molecular docking

Naringenin’s 3D structure was generated (ChemBioDraw/MMFF94) and docked with target proteins (RCSB PDB structures) using AutoDock Vina. Docking results were visualized with PyMOL and Maestro; 2D interactions were mapped using MOE.

### 2.18 Chromatin immunoprecipitation (ChIP)

VSMCs (∼1 × 10^7^) were cross-linked with 1% formaldehyde, quenched with glycine, and chromatin was sonicated to 200–1,000 bp. Chromatin immunoprecipitation (ChIP) was performed in VSMCs expressing FLAG-tagged p53. Immunoprecipitation was performed with anti-FLAG or IgG control, and DNA was purified for qPCR analysis of *TOP2A* promoter enrichment. Primer sequences are in Supplementary Table S4.

### 2.19 Dual-luciferase reporter assay

VSMCs were co-transfected with firefly luciferase reporters containing WT or mutant *TOP2A* promoter and Renilla plasmids. After 48 h, luciferase activities were measured and normalized (Promega Dual-Luciferase System). Each experiment was performed in triplicate.

### 2.20 Statistical analysis

Data are presented as mean ± SEM unless otherwise indicated. Differences between two groups were analyzed using unpaired two-tailed Student’s t-tests, and multiple-group comparisons were performed using one-way ANOVA with appropriate *post hoc* tests. A p value <0.05 was considered statistically significant. Kaplan-Meier analysis was used to assess AVF failure-free survival, with follow-up time measured in weeks, and group differences were compared using the log-rank test. Given the limited sample size, this analysis was considered exploratory. All statistical analyses were performed using GraphPad Prism 9.

## 3 Results

### 3.1 Vascular calcification is closely associated with senescence-associated changes

**FIGURE 1:**
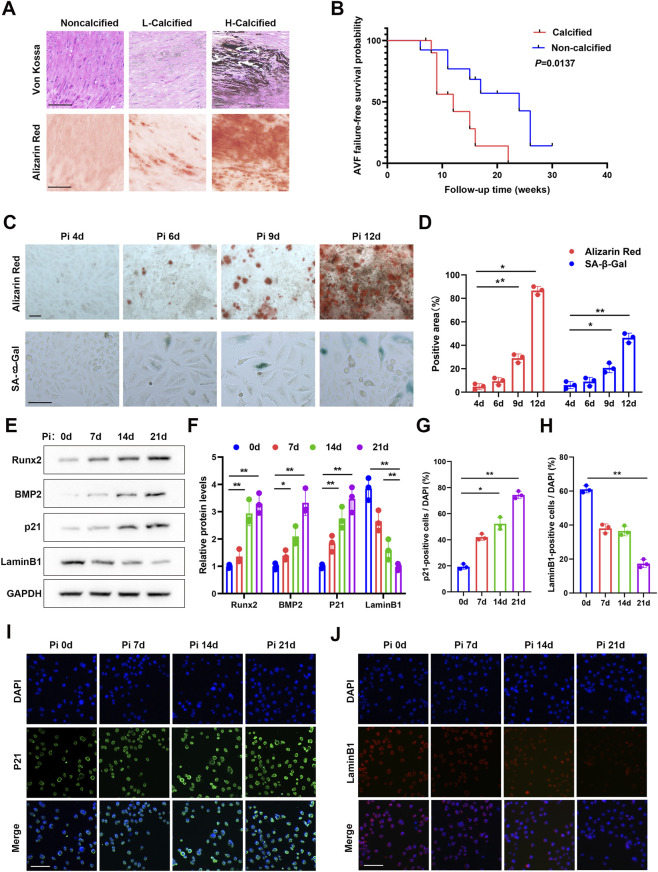
Positive association between vascular calcification and senescence-associated changes. Human vascular specimens were classified by Von Kossa and ARS staining **(A)**, and exploratory Kaplan-Meier analysis showed worse AVF failure-free survival in the calcified group **(B)**. *In vitro*, phosphate-induced calcification in VSMCs was accompanied by increased SA-β-Gal staining, changes in osteogenic and senescence-related proteins, and corresponding immunofluorescence alterations in p21 and LaminB1 in parallel time-course experiments **(C–J)**. Scale bars are as indicated. *p < 0.05; **p < 0.01.

Based on Von Kossa and ARS staining, radial artery specimens from patients with CKD5 were classified into non-calcified, lightly calcified, and heavily calcified groups (Figure 1A). Given the limited sample size, the lightly calcified and heavily calcified groups were further combined into a single calcified group for exploratory follow-up analysis. Patients were followed on a weekly basis for up to 32 weeks. Kaplan-Meier analysis suggested that, compared with the non-calcified group, the calcified group had worse AVF failure-free survival and a higher risk of AVF failure (Figure 1B). *In vitro*, progressive calcium deposition was accompanied by increased SA-β-Gal positivity (Figures 1C,D), increased expression of osteogenic and senescence-related markers (Figures 1E,F), and corresponding immunofluorescence changes in p21 and LaminB1 (Figures 1G–J). However, the clinical observations should be interpreted cautiously because of the limited sample size and lack of multivariable adjustment.

### 3.2 Naringenin is identified as a candidate compound for vascular calcification

**FIGURE 2:**
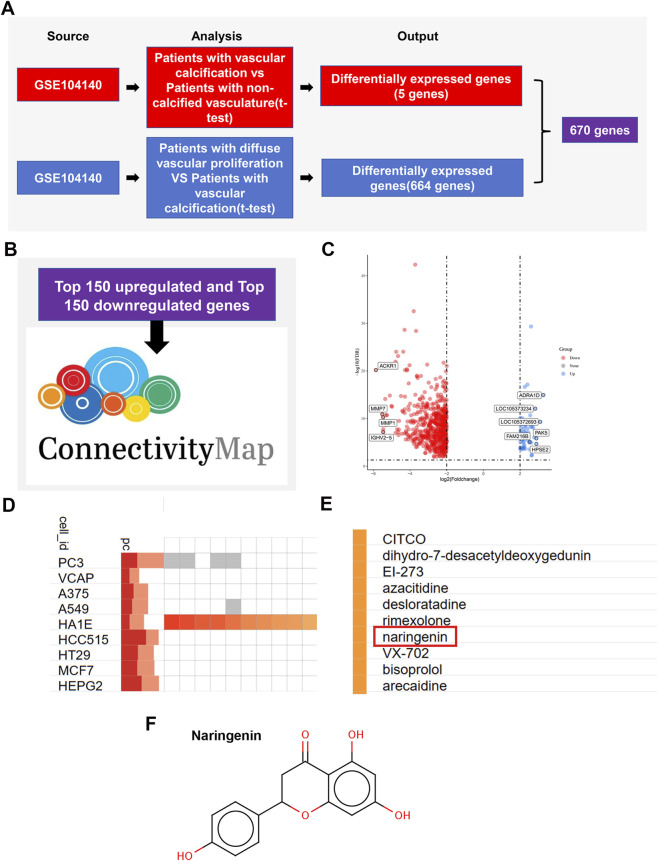
Naringenin identified as a candidate compound for vascular calcification. Analysis of GEO datasets comparing vascular calcification and control groups identified 670 differentially expressed genes **(A)**. Given the limited clinical sample size and inter-patient heterogeneity, these transcriptomic results were used as an exploratory screening step. The top 150 upregulated and downregulated genes were queried in the CMAP database, yielding a panel of candidate small molecules **(B,C)**. Compound–cell line association analysis highlighted naringenin as a candidate compound with a potentially relevant inverse transcriptional signature **(D,E)**, and its chemical structure is shown **(F)**.

Differential expression analysis of GEO dataset GSE104140 yielded 670 dysregulated genes (Figure 2A). Given the limited clinical sample size, inter-patient heterogeneity, and the relatively stringent screening thresholds, the transcriptomic results were interpreted as an exploratory screening step and were further integrated with CMap analysis and downstream experimental validation. The top 150 upregulated and top 150 downregulated DEGs were submitted to the Connectivity Map (CMap) database to identify small-molecule drugs that produce an opposite gene expression signature (Figure 2B). Volcano plot analysis further highlighted the significant distribution of these DEGs (Figure 2C). Compound–cell line association revealed several candidate small molecules, among which naringenin (Nar) was strongly implicated (Figures 2D,E). The chemical structure of naringenin was confirmed (Figure 2F). These *in silico* findings nominated naringenin as a candidate small molecule for further validation in vascular calcification.

### 3.3 Naringenin attenuates calcification and senescence-associated changes in VSMCs *in vitro*

**FIGURE 3:**
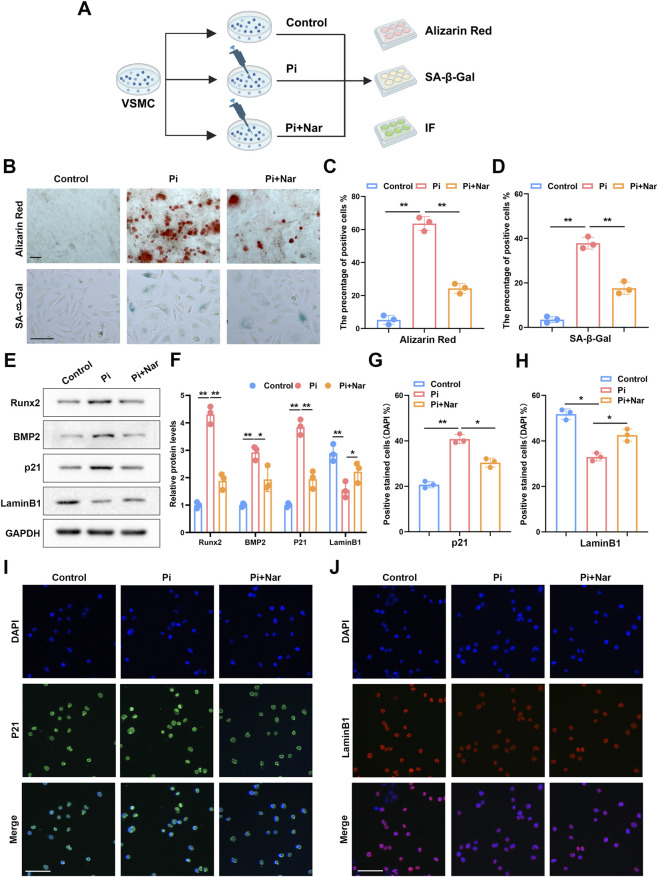
Naringenin attenuates vascular smooth muscle cell calcification and senescence-associated changes. In a phosphate-induced calcification model, the effects of naringenin on senescence-associated changes and calcification were assessed **(A)**. Alizarin red and SA-β-Gal staining showed that naringenin markedly reduced calcium deposition and senescence-associated changes **(B–D)**. Western blotting demonstrated decreased expression of osteogenic proteins (Runx2, BMP2) and the senescence marker p21, with increased LaminB1 expression **(E,F)**. Immunofluorescence confirmed a reduction in p21-positive cells and restoration of LaminB1 following naringenin treatment **(G–J)**. Scale bar: 100 µm *p < 0.05; **p < 0.01.

In the phosphate-induced VSMC calcification model, naringenin treatment markedly reduced Alizarin Red–positive calcified nodules and SA-β-Gal–positive cells (Figures 3A,B). Quantitative analysis confirmed significant decreases in calcification area and SA-β-Gal positivity (Figures 3C,D). Western blot demonstrated that naringenin downregulated Runx2, BMP2, and p21, while restoring LaminB1 expression (Figures 3E,F). Immunofluorescence results were consistent, showing reduced p21 and preserved LaminB1 expression under naringenin treatment (Figures 3G,H). Collectively, these results demonstrate that naringenin effectively suppresses VSMC calcification and attenuates senescence-associated changes *in vitro*.

### 3.4 Naringenin alleviates vascular calcification and senescence-associated changes *in vivo*

**FIGURE 4:**
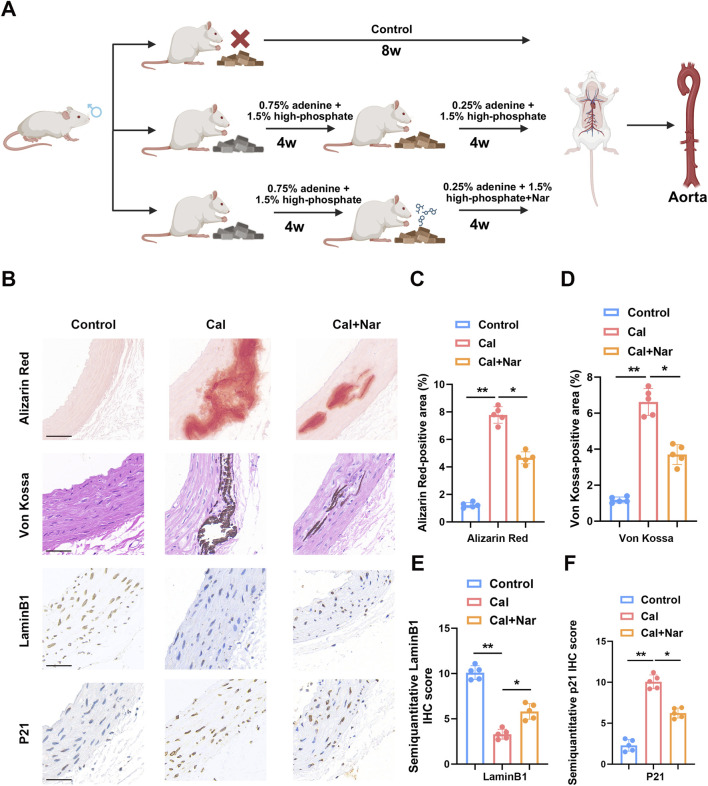
Naringenin suppresses vascular calcification and senescence-associated changes in vivo. In an adenine/high-phosphate-induced CKD-associated vascular calcification rat model, rats were treated with naringenin from week 5 to week 8, and aortic tissues were harvested at the end of week 8 for analysis **(A)**. Animal data are presented as mean ± SD; each dot represents one rat. Naringenin significantly reduced Alizarin Red S and Von Kossa staining positivity and alleviated senescence-associated changes in the aorta. Immunohistochemistry further demonstrated reduced p21 expression and restoration of Lamin B1 levels after naringenin treatment **(B–F)**. Scale bar: 50 μm *p < 0.05; **p < 0.01.

We then tested naringenin in an *in vivo* model of CKD-associated vascular calcification (Supplementary Figure S1A,B) (Figure 4A). In the rat model fed with high phosphate/adenine diet, the aortas developed significant calcification, as evidenced by robust calcium deposits on Alizarin Red S and Von Kossa staining in the model group. Naringenin administration greatly attenuated this vascular calcification: aortas from naringenin-treated rats showed far fewer calcium deposits compared to untreated CKD model rats (Figures 4B–D). Furthermore, immunohistochemical analysis of aortic sections revealed that calcified vessels from CKD rats had reduced Lamin B1 expression and elevated p21 expression, consistent with enhanced senescence-associated changes. Importantly, naringenin treatment largely reversed these molecular changes *in vivo* – maintaining Lamin B1 levels and lowering p21 levels in the aortic tissue (Figures 4E,F). These *in vivo* findings confirm that naringenin confers a dual protective effect against vascular calcification and senescence-associated alterations at the tissue level, in agreement with the *in vitro* results.

### 3.5 Transcriptomic analysis suggests involvement of the p53 pathway and TOP2A

**FIGURE 5:**
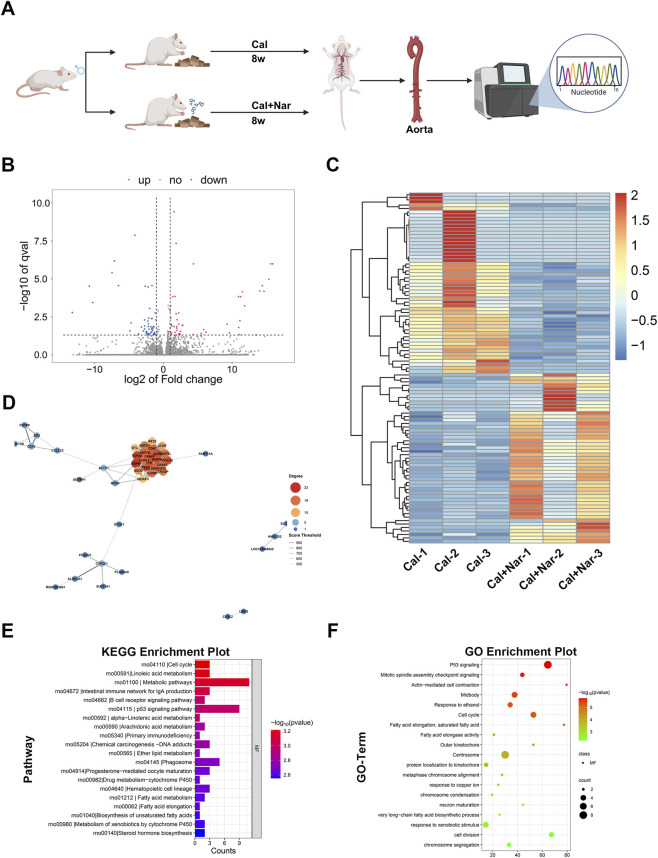
Naringenin modulates the p53 signaling pathway and downstream effector TOP2A. RNA sequencing comparing aortic tissues from the CKD-VC group and the CKD-VC + naringenin group identified significantly differentially expressed genes (|log_2_FC| > 1, P < 0.05) **(A)**. Volcano plots and heatmaps showed marked transcriptional changes **(B,C)**. Network correlation analysis indicated potential downstream effectors **(D)**. KEGG and GO enrichment analyses revealed enrichment in the p53 signaling pathway and related processes involving cell cycle and apoptosis regulation **(E,F)**.

RNA sequencing of rat aortic tissues collected at the end of week 8 revealed distinct DEGs between the calcified and naringenin-treated groups (Figures 5A,B). Heatmap visualization demonstrated clear clustering between groups (Figure 5C), and gene interaction network analysis identified key functional modules (Figure 5D). Although some degree of within-group variability was observed, this likely reflects biological heterogeneity among animals and is not unexpected given the limited sample size of the RNA-seq analysis. KEGG enrichment analysis indicated that DEGs were predominantly enriched in cell cycle, metabolic pathways, and the p53 signaling pathway (Figure 5E). GO analysis further implicated biological processes such as DNA repair, chromosome segregation, and cell division within the p53 regulatory framework (Figure 5F). These transcriptomic data suggest that naringenin may exert its protective effects through the p53 signaling pathway, with TOP2A as a potential downstream effector.

### 3.6 Naringenin is associated with suppression of p53 signaling and relief of p53-mediated transcriptional repression of TOP2A

**FIGURE 6:**
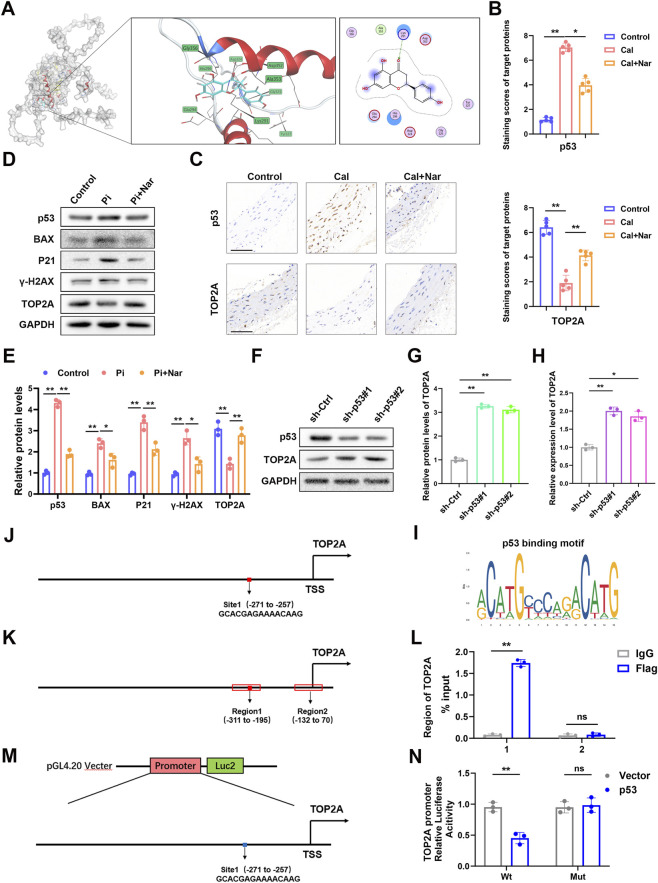
Naringenin is associated with suppression of p53 signaling and relief of p53-mediated transcriptional repression of TOP2A. Molecular docking suggested a potential interaction between naringenin and p53 **(A)**. Immunohistochemistry showed decreased p53 and increased TOP2A levels in aortic tissues after naringenin treatment; scale bar: 50 µm **(B,C)**. Western blotting confirmed downregulation of p53, BAX, p21, and γ-H2AX, with upregulation of TOP2A **(D,E)**. In VSMCs, p53 knockdown significantly increased TOP2A expression **(F–H)**. JASPAR analysis revealed p53 binding motifs in the TOP2A promoter region **(I,J)**, supported by ChIP-PCR **(K,L)**. Dual-luciferase reporter assays demonstrated that p53 overexpression suppressed TOP2A promoter activity, whereas mutation of the binding site abolished this effect **(M,N)**. Scale bar: 100 μm; *p < 0.05; **p < 0.01.

Molecular docking suggested a potential interaction between naringenin and p53; however, this *in silico* result alone does not establish direct binding or direct inhibition (Figure 6A). Immunohistochemistry revealed decreased p53 and increased TOP2A expression in naringenin-treated rat aortas (Figures 6B,C). Western blot analysis showed reduced expression of p53 and its downstream effectors, including BAX, p21, and γ-H2AX, whereas TOP2A expression was increased after naringenin treatment (Figures 6D,E). *In vitro* knockdown of p53 further supported a negative regulatory effect of p53 on TOP2A expression at both the protein and mRNA levels (Figures 6F–H). JASPAR analysis predicted p53 binding sites within the TOP2A promoter region (Figures 6I,J), which were further supported by ChIP-PCR (Figures 6K,L). Dual-luciferase reporter assays demonstrated that p53 overexpression suppressed TOP2A promoter activity, whereas mutation of the predicted binding site abolished this effect (Figures 6M,N). Together, these findings support that naringenin is associated with suppression of p53 signaling, accompanied by relief of p53-mediated transcriptional repression of TOP2A and restoration of TOP2A expression.

### 3.7 The p53/TOP2A axis contributes substantially to the anti-calcification effect and the accompanying attenuation of senescence-associated changes

**FIGURE 7:**
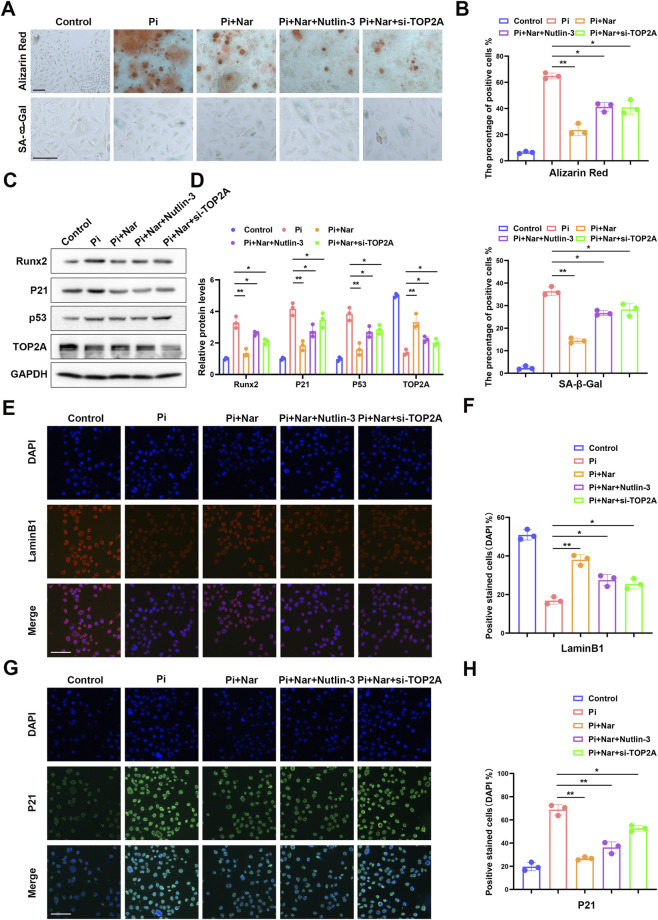
Naringenin attenuates vascular smooth muscle cell calcification and accompanying senescence-associated changes, at least in part, via the p53/TOP2A axis. To further elucidate the mechanism, the p53 activator Nutlin-3 and TOP2A-targeting siRNA (si-TOP2A) were applied **(A)**. Alizarin Red S and SA-β-Gal staining revealed that either p53 activation or TOP2A knockdown significantly attenuated the protective effects of naringenin **(B)**. Western blotting showed that Nutlin-3 and si-TOP2A partially reversed naringenin-mediated regulation of Runx2, p21, p53, and TOP2A **(C,D)**. Immunofluorescence confirmed that disruption of the p53/TOP2A axis attenuated naringenin-induced LaminB1 restoration and p21 reduction **(E–H)**. Scale bar: 100 μm; *p < 0.05; **p < 0.01.

To further examine whether the protective effects of naringenin were mediated, at least in part, through the p53/TOP2A axis, we performed rescue experiments in calcifying VSMCs. In naringenin-treated cultures, activation of p53 with Nutlin-3 or knockdown of TOP2A using siRNA significantly attenuated the protective effects of naringenin. Compared with the Pi + Nar group, cells exposed to naringenin plus Nutlin-3 or naringenin plus si-TOP2A showed increased calcification and senescence-associated changes, as evidenced by more extensive Alizarin Red staining and higher SA-β-Gal positivity (Figures 7A,B). Western blot analysis further showed that Nutlin-3 treatment or TOP2A silencing partially reversed the naringenin-mediated regulation of p53, p21, and TOP2A (Figures 7C,D). Immunofluorescence confirmed that the naringenin-induced restoration of LaminB1 and suppression of p21 were significantly diminished after these interventions (Figures 7E–H). These findings support that the p53/TOP2A axis mediates, at least in part, the anti-calcific effect of naringenin and is associated with attenuation of senescence-associated changes. However, the present data do not establish that osteogenic differentiation is mediated exclusively through modulation of senescence-associated changes. Nor do they determine whether TOP2A knockdown alone is sufficient to induce osteogenic marker expression independently of senescence-associated changes.

## 4 Discussion

Chronic kidney disease (CKD) is a major global health concern associated with high cardiovascular morbidity and mortality (Cockwell and Fisher, 2020). By 2040, CKD is projected to rank among the top five causes of death worldwide (Foreman et al., 2018). CKD patients have an elevated risk of premature cardiovascular disease (CVD), and within this context, vascular calcification (VC) stands out as one of the strongest predictors of adverse cardiovascular outcomes (Marreiros et al., 2022). Unfortunately, early diagnosis of VC in CKD remains difficult because of its multifactorial, complex pathogenesis (Ding et al., 2023). VC can occur in both the intimal and medial layers of arteries and is driven by diverse, interacting pathological mechanisms even in the initial stages (Kaur and Singh, 2022). It is a prominent feature in a spectrum of conditions including atherosclerosis (intimal calcification), aortic valve stenosis, aging, diabetes, and notably CKD (Gallo Marin et al., 2023; Lanzer et al., 2021). Arterial stiffening and calcification are well-recognized hallmarks of vascular aging and contribute significantly to cardiovascular morbidity and mortality (Redheuil et al., 2014).

Senescence-associated changes have emerged as an important contributor linking CKD to VC and CVD (Stojanović et al., 2020). Cells with senescence-associated changes may lose proliferative capacity while remaining metabolically active and exhibit characteristic changes: for example, they develop enlarged cell size, express senescence-associated β-galactosidase (SA-β-gal) activity, and upregulate cell-cycle inhibitors like p53 and p21 (Gorgoulis et al., 2019). These changes promote vascular stiffness, luminal narrowing, and even occlusion, thereby elevating the risk of cardiovascular events (Mehdizadeh et al., 2022). As early as the work of Virchow, pathological alterations in the arteries of CKD patients were noted to resemble intimal calcification changes observed in the elderly (Virchow, 1989). Within the vascular media, vascular smooth muscle cells (VSMCs) are the predominant functional cells, and their osteogenic phenotypic switch represents a central step in medial calcification (Durham et al., 2018). Importantly, VSMCs with senescence-associated changes are more prone to undergo osteogenic transdifferentiation, accelerating calcium deposition (Nakano-Kurimoto et al., 2009). Our study confirmed this concept, demonstrating a positive correlation between senescence-associated changes in VSMCs and the extent of calcification.

Strategies to modulate senescence-associated pathways and prevent CKD-associated cardiovascular complications have long been a research focus. In the present study, we screened differentially expressed genes in VC using public datasets and identified potential therapeutic agents through Connectivity Map (cMAP) analysis. This led to the discovery of naringenin, a natural citrus flavonoid, as a promising candidate compound. Naringenin has broad biological activities and has been implicated in arthritis, intestinal inflammation, cardiovascular disease, diabetes, neurodegenerative disorders, and cancer prevention (Zeng et al., 2018; Salehi et al., 2019). It exhibits strong antioxidant properties and anti-inflammatory effects, known to inhibit pro-inflammatory mediators such as NF-κB, cyclooxygenase-2 (COX-2), and interleukin-1β, and it can improve lipid profiles by lowering total and LDL cholesterol (Chen et al., 2021; Park et al., 2012; Kong et al., 2020). Additionally, naringenin can activate the AMPK pathway, thereby promoting autophagy, maintaining intracellular homeostasis, and modulating senescence-associated changes (Chen et al., 2022). Consistent with these known properties, our experimental results showed that naringenin significantly reduced phosphate-induced senescence-associated changes in VSMCs and calcification *in vitro*, and mitigated vascular calcification *in vivo*. These findings suggest that naringenin can play a critical role in preventing CKD-associated VC, likely through its combined antioxidative, anti-inflammatory, and anti-calcific actions, accompanied by attenuation of senescence-associated changes.

Within the vascular system, aberrant activation of the p53 pathway is closely linked to both vascular calcification and senescence-associated changes. On one hand, p53 upregulates downstream effectors such as p21, inducing VSMC cell cycle arrest and senescence-associated phenotypes (e.g., SA-β-gal positivity), which contribute to vascular aging (Fang et al., 2024). On the other hand, p53 signaling promotes VSMC apoptosis and osteogenic differentiation, accelerating mineral deposition within the vascular wall (Liu et al., 2013). Previous studies have shown that under high phosphate burden combined with inflammatory stimuli, VSMCs undergo significant p53-dependent senescence-associated changes and calcification, which can be inhibited in a dose-dependent manner by agents that attenuate senescence-associated changes, such as resveratrol. Similarly, uremic toxins induce senescence-associated changes and calcification via oxidative stress–mediated upregulation of p53/p21 (Fang et al., 2024). Collectively, these findings underscore the potential role of p53-associated stress responses in the initiation and progression of VC. Although our data consistently showed that naringenin reduced p53 expression/activity and relieved p53-mediated repression of TOP2A, the precise upstream mechanism remains unresolved. Molecular docking provides only preliminary *in silico* support and is insufficient to establish direct target engagement. Given that calcification-related stress is closely linked to oxidative stress and DNA damage signaling, it is plausible that naringenin suppresses p53 indirectly through modulation of ROS accumulation, DNA damage response pathways, or p53 stability. These possibilities require further investigation.

TOP2A (DNA topoisomerase IIα) has emerged as an important downstream effector in the p53 regulatory network, with significant relevance to cell proliferation and cellular stress responses. During sustained cellular stress, persistent activation of p53 (often together with the p16/INK4a/RB pathway) leads to assembly of the DREAM transcriptional repressor complex, which silences a host of proliferation-promoting and DNA repair genes, including TOP2A (Kandhaya-Pillai et al., 2023). In fact, sustained p53 activity has been shown to directly suppress TOP2A transcription, resulting in reduced TOP2A protein levels, diminished cell proliferative capacity, and impaired DNA repair – all of which consolidate persistent growth arrest (Kandhaya-Pillai et al., 2023). However, TOP2A should be interpreted with caution in the present study. Our findings suggest that TOP2A functions as an important downstream mediator linked to the protective effects of naringenin and the attenuation of senescence-associated changes, but they do not support TOP2A as a validated independent therapeutic target. Therefore, TOP2A is more appropriately regarded as a mechanistically relevant downstream effector within the protective pathway modulated by naringenin.

Our study further elucidated the molecular mechanism of naringenin: it attenuated hyperactivation of the p53 pathway, thereby relieving p53-mediated transcriptional repression of TOP2A and restoring TOP2A expression. By downregulating p53 and upregulating TOP2A, naringenin preserved VSMC proliferative and DNA repair capacity under calcifying stress. This dual effect not only reduced senescence-associated phenotypes but also limited osteogenic transdifferentiation, thereby decreasing vascular calcification. However, these processes should not be considered fully interchangeable. While our findings support a close association between attenuation of senescence-associated changes and reduced osteogenic differentiation, they do not definitively establish that these pathways are mechanistically inseparable. The upstream mechanism by which naringenin suppresses p53 remains unclear, and molecular docking alone does not demonstrate direct target engagement. TOP2A should be regarded as a downstream mediator rather than a validated therapeutic target. In addition, the relationship between senescence-associated changes and osteogenic/calcific phenotypes was not fully disentangled, and SASP-related factors were not systematically assessed. RNA-seq and human cohort analyses should be considered exploratory because of the limited sample size and lack of multivariable adjustment. Moreover, renal functional parameters were not systematically evaluated in the animal model.

**FIGURE 8:**
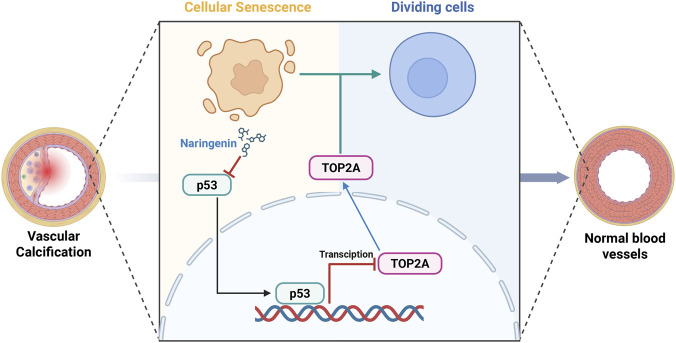
Proposed mechanism of naringenin in vascular calcification and senescence-associated changes. Schematic illustration showing that naringenin attenuates vascular calcification and accompanying senescence-associated changes, at least in part, through modulation of the p53/TOP2A axis. The diagram was created with BioRender.com.

In conclusion, our findings suggest that naringenin inhibits vascular calcification, at least in part, through suppression of p53 signaling and restoration of TOP2A (Figure 8). This protective effect is accompanied by attenuation of senescence-associated changes. However, the upstream mechanism of p53 suppression, the precise role of TOP2A, and the causal relationship between senescence-associated changes and osteogenic differentiation require further investigation.

## Data Availability

The datasets presented in this study can be found in online repositories. The names of the repository/repositories and accession number(s) can be found in the article/Supplementary Material.
