# Supplementary material for: Naringenin inhibited vascular calcification and attenuated senescence-associated changes through the p53/TOP2Aaxis
Source: Front Pharmacol. 2026 May 8;17:1785797. doi: 10.3389/fphar.2026.1785797 (PMC13194564; doi:10.3389/fphar.2026.1785797)
Supplement: Supplementary file 1 [file Table1.docx]

**Supplementary Table 1** Primer sequences of relevant genes used.

| **Gene** | **Sequence（5′ ->3′）** | |
| --- | --- | --- |
| P53 | Forward Primer | AAGTCTAGAGCCACCGTCCA |
|  | Reverse Primer | CAGTCTGGCTGCCAATCCA |
| TOP2A | Forward Primer | TGTCACCATTGCAGCCTGT |
|  | Reverse Primer | TGTCTGGGCGGAGCAAAATA |
| GAPDH | Forward Primer | CTCCAAAATCAAGTGGGGCG |
|  | Reverse Primer | TGGTTCACACCCATGACGAA |

**Supplementary Table 2** Target sequences of relevant genes used.

| **Gene** | **Targeting sequences** |
| --- | --- |
| sh-P53#1 | 5′- GCTCGACGCTAGGATCTGAC-3′ |
| sh-P53#2 | 5′- TGACACGCTTCCCTGGATTG-3′ |
| si-TOP2A | 5′- ACCAATGTAGGTGTCTGGGC-3′ |

**Supplementary Table 3** Antibodies and dilution ratio of relevant proteins used.

| **Gene** | **Brand** | **Catalog number** | **WB** | **IP** | **IHC** | **IF** |
| --- | --- | --- | --- | --- | --- | --- |
| P53 | Abcam | Ab131442 | 1:1000 | 1:50 | 1:100 |  |
| TOP2A | Cell Signaling | 12286 | 1:1000 | 1:100 | 1:1000 |  |
| Flag | Proteintech | 20543-1-AP |  | 1:50 |  |  |
| IgG | Proteintech | 30000-0-AP |  | 1:50 |  |  |
| RUNX2 | Proteintech | 20700-1-AP | 1:1000 |  |  |  |
| BMP2 | Proteintech | 66383-1-Ig | 1:1000 |  |  |  |
| GAPDH | Cell Signaling | 5174 | 1:1000 |  |  |  |
| Lamin B1 | Proteintech | 12987-1-AP | 1:1000 |  | 1:200 | 1:100 |
| P21 | Abcam | ab109520 | 1:1000 |  | 1:200 | 1:100 |
| BAX | Abcam | ab32503 | 1:1000 |  |  |  |
| γ-H2AX | Proteintech | 10856-1-AP | 1:1000 |  |  |  |

**Supplementary Table 4** Primer sequences of ChIP-PCR.

| **Promoter** | **Region** | **Primers** | **Sequence( 5′ to 3′)** |
| --- | --- | --- | --- |
| TOP2A | 1 | Forward primer | CCCCTTCCCGCTTCCAAA |
|  |  | Reverse primer | AGGCACCACTCGGCGTCAT |
|  | 2 | Forward primer | ACTCAGCCGTTCATAGGTGG |
|  |  | Reverse primer | GCGACTAAACAGGCAGGAC |
